# *Enterococcus faecalis* Polymicrobial Interactions Facilitate Biofilm Formation, Antibiotic Recalcitrance, and Persistent Colonization of the Catheterized Urinary Tract

**DOI:** 10.3390/pathogens9100835

**Published:** 2020-10-13

**Authors:** Jordan R. Gaston, Marissa J. Andersen, Alexandra O. Johnson, Kirsten L. Bair, Christopher M. Sullivan, L. Beryl Guterman, Ashely N. White, Aimee L. Brauer, Brian S. Learman, Ana L. Flores-Mireles, Chelsie E. Armbruster

**Affiliations:** 1 Department of Medicine, Jacobs School of Medicine and Biomedical Sciences, State University of New York at Buffalo, NY 14203, USA; jgaston2@buffalo.edu (J.R.G.); cs285@buffalo.edu (C.M.S.); laurengu@buffalo.edu (L.B.G.); 2 Department of Biological Sciences, College of Science, Notre Dame University, IN 15701, USA; mander40@nd.edu; 3 Department of Microbiology and Immunology, Jacobs School of Medicine and Biomedical Sciences, State University of New York at Buffalo, NY 14203, USA; aj77@buffalo.edu (A.O.J.); klbair@buffalo.edu (K.L.B.); ashleywh@buffalo.edu (A.N.W.); albrauer@buffalo.edu (A.L.B.); bslearma@buffalo.edu (B.S.L.)

**Keywords:** proteus mirabilis, enterococcus faecalis, polymicrobial, biofilm, catheter, urinary tract infection

## Abstract

Indwelling urinary catheters are common in health care settings and can lead to catheter-associated urinary tract infection (CAUTI). Long-term catheterization causes polymicrobial colonization of the catheter and urine, for which the clinical significance is poorly understood. Through prospective assessment of catheter urine colonization, we identified *Enterococcus faecalis* and *Proteus mirabilis* as the most prevalent and persistent co-colonizers. Clinical isolates of both species successfully co-colonized in a murine model of CAUTI, and they were observed to co-localize on catheter biofilms during infection. We further demonstrate that *P. mirabilis* preferentially adheres to *E. faecalis* during biofilm formation, and that contact-dependent interactions between *E. faecalis* and *P. mirabilis* facilitate establishment of a robust biofilm architecture that enhances antimicrobial resistance for both species. *E. faecalis* may therefore act as a pioneer species on urinary catheters, establishing an ideal surface for persistent colonization by more traditional pathogens such as *P. mirabilis*.

## 1. Introduction

Placement of an indwelling urinary catheter is a common medical procedure in health care settings, and is estimated to occur during treatment of 60% of critically ill patients, 20% of patients in medical and surgical intensive care units, and 5–22% of residents in long-term care facilities, such as nursing homes [1,2,3,4,5,6,7,8]. The presence of an indwelling catheter facilitates bacterial colonization of the urine (bacteriuria), partly due to development of a conditioning film of host proteins that provide bacteria with an ideal substrate for attachment [9,10,11]. For each day that a urinary catheter is in place, there is a 3–8% incidence of bacteriuria, and long-term catheterization (> 28 days) typically results in continuous bacteriuria and symptomatic CAUTI [1,12]. Colonizing bacteria also frequently persist even after catheter changes and antimicrobial treatment due to the development of bacterial biofilms on the catheter itself and intracellular bacterial communities within bladder epithelial cells [13,14,15,16]. 

Prior studies of catheter-associated bacteriuria revealed that early colonization typically involves *Escherichia coli, Enterococcus* spp., *Pseudomonas aeruginosa,* coagulase-negative *Staphylococcus* spp., yeast spp., *Klebsiella* spp., and *Enterobacter* spp. (see [17,18] for review). During long-term catheterization, some of the early colonizers remain prevalent, such as *Enterococcus* spp., *E. coli,* and *P. aeruginosa*, while other organisms not often encountered during short-term catheterization become more common, including *Proteus mirabilis, Providencia* spp., and *Morganella morganii.* The likelihood of having multiple bacterial species present at once also increases during prolonged catheterization. In fact, the majority of nursing home residents with long-term indwelling catheters exhibit polymicrobial bacteriuria and often have polymicrobial infection [1,2,15,19,20,21,22,23,24,25,26]. For instance, in a cohort of nursing homes in southeast Michigan, 31% of recent CAUTIs for which antimicrobials were prescribed were polymicrobial, and the most common causes of polymicrobial CAUTI were *Enterococcus faecalis* and *P. mirabilis* [19].

Despite the high prevalence of polymicrobial CAUTI, the consequences of polymicrobial urine colonization are poorly understood. In other infection settings, polymicrobial interactions have been demonstrated to either increase or attenuate persistence and systemic dissemination, depending on the combination of organisms being studied and the type of infection [27]. In the context of urinary tract infection, only a handful of studies have explored the consequences of polymicrobial colonization, the majority of which have indicated that polymicrobial interactions increase colonization and persistence [11,28,29,30,31,32,33]. We recently determined that factors secreted by *E. faecalis* can enhance the pathogenic potential of *P. mirabilis* by promoting urease activity and cytotoxicity, and that co-infection of *E. faecalis* with *P. mirabilis* increases tissue damage and the incidence of urolithiasis and bacteremia during experimental CAUTI [34]. Thus, the presence of both *E. faecalis* and *P. mirabilis* at a high bacterial burden can be detrimental to the host, and further exploration of the factors that promote colonization by these species may elucidate new targets for reducing the risk of developing CAUTI and severe sequelae. 

This study details the prevalence and duration of *E. faecalis* and *P. mirabilis* bacteriuria in a cohort of nursing home residents over the course of up to 30 weeks, as well as their co-localization on catheters during experimental infection in a mouse model of CAUTI and during biofilm formation in vitro. Our results demonstrate that: 1) *E. faecalis* and *P. mirabilis* frequently and persistently co-colonize catheterized individuals, 2) *E. faecalis* facilitates stable bladder and catheter colonization by *P. mirabilis,* and 3) contact-dependent interactions between these pathogens result in a dramatic increase in biofilm biomass and architecture complexity that provides protection to both organisms from antimicrobials empirically used to treat CAUTI. These data indicate that interspecies interactions between bacteria that commonly colonize the catheterized urinary tract can result in a mutualistic pathogenic phenotype benefiting both species, at a potential detriment to the host. 

## 2. Results

### 2.1. Proteus mirabilis and Enterococcus faecalis Persistently Co-colonize in Catheterized Nursing Home Residents

We recently conducted a prospective assessment of the etiology and dynamics of asymptomatic bacteriuria in catheterized individuals, in which *E. faecalis* and *P. mirabilis* were identified as the most prevalent and persistent colonizing organisms and a common combination from polymicrobial samples [35]. We therefore further examined the weekly dynamics of *E. faecalis* and *P. mirabilis* co-colonization in each study participant. Of 19 study participants, 18 (95%) were colonized by *E. faecalis* during at least one study visit, 11 (58%) were colonized by *P. mirabilis* during at least one study visit, and 9 (47%) exhibited co-colonization by *E. faecalis* and *P. mirabilis* during at least one study visit [35]. Of the participants who did not exhibit *E. faecalis* and *P. mirabilis* co-colonization, 8 (80%) were colonized by *E. faecalis* without *P. mirabilis,* 1 was colonized by *P. mirabilis* without *E. faecalis,* and 1 was colonized by both species but never at the same time. 

**Figure 1:**
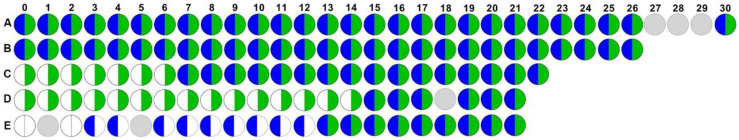
*Enterococcus faecalis* and *Proteus mirabilis* persistently co-colonize during long-term catheterization. Data represent the colonization status of *E. faecalis* (right half, green) and *P. mirabilis* (left half, blue) in weekly urine cultures from baseline (0) to 30 weeks for each of five study participants (labeled A–E). White semi-circles indicate that a urine sample was collected but lacked *P. mirabilis* or *E. faecalis,* and gray circles indicate that a urine sample was not collected at that study visit.

Of the nine participants who exhibited co-colonization, five completed at least 21 weeks of follow up, which allowed for prospective assessment of the dynamics and persistence of *E. faecalis* and *P. mirabilis* co-colonization (Figure 1). In this subgroup of five participants, two were co-colonized for the entire duration of follow up (Figure 1A and B, co-colonized for 30 and 26 weeks, respectively), two were colonized with *E. faecalis* for the duration of the study but acquired *P. mirabilis* and remained persistently co-colonized for the remainder of follow up (Figure 1C and D, co-colonized for 16 and 7 weeks, respectively), and one was initially negative for both species but acquired *P. mirabilis* during week 3 and *E. faecalis* during week 13 and remained persistently co-colonized for the remainder of follow up (Figure 1E, co-colonized for 9 weeks). Taken together, these data demonstrate that *E. faecalis* and *P. mirabilis* frequently and persistently co-colonize catheterized nursing home residents, and may indicate a synergistic role for *E. faecalis* in facilitating *P. mirabilis* colonization and persistence. Thus, the interaction of these organisms and how they persist on urinary catheters warrant further investigation. 

### 2.2. Enterococcus faecalis and Proteus mirabilis Co-localize during Experimental CAUTI

**Figure 2:**
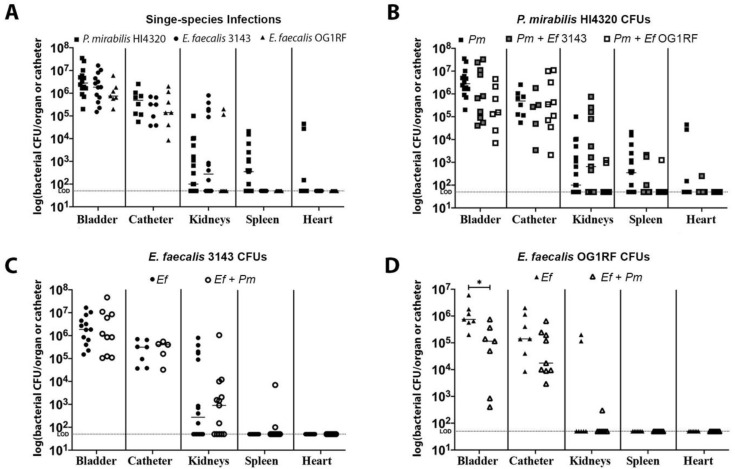
*E. faecalis* and *P. mirabilis* co-colonize the catheterized urinary tract during experimental infection. The 24 h mouse model of CAUTI using *E. faecalis* 3143 (circles), *E. faecalis* OG1RF (triangles), and *P. mirabilis* HI4320 (squares) either in a single-infection (black symbols) or co-infection (open symbols). Each symbol represents the log_10_ CFU/organ or catheter from an individual mouse. (**A**) Bacterial burden of each bacterial species during single-species infection. (**B**) *P. mirabilis* colonization during single-species infection compared to co-infection with *E. faecalis* 3143 or *E. faecalis* OG1RF. (**C**) *E. faecalis* 3143 colonization during single-species infection compared to co-infection with *P. mirabilis* HI4320. (**D**) *E. faecalis* OG1RF colonization during single-species infection compared to co-infection with *P. mirabilis* HI4320. Dashed lines indicate limit of detection, and error bars indicate the median. Mann–Whitney *U* test was used to compare groups. Statistical significance is represented by *P < 0.05.

**Figure 3:**
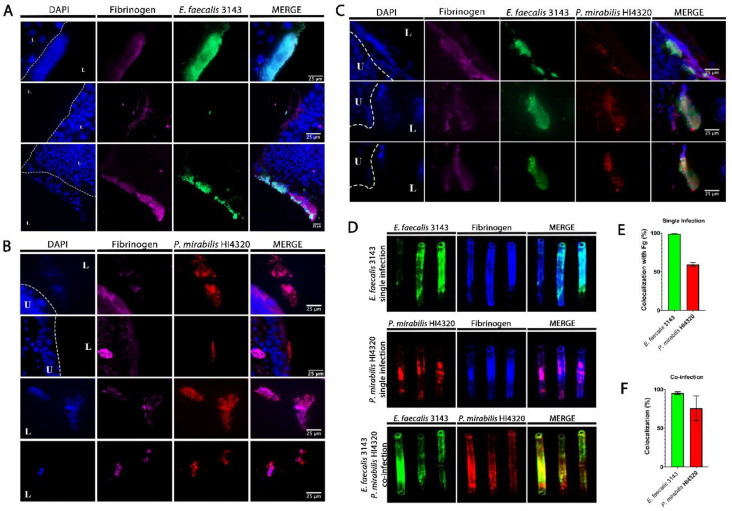
*E. faecalis* co-localizes with fibrinogen and *P. mirabilis* co-localizes with *E. faecalis* in the catheterized bladder and on catheter segments. (**A–C**) Representative sections of bladders removed 24 hpi from mice that were catheter-implanted and infected with *E. faecalis* 3143 (**A**), *P. mirabilis* HI4320 (**B**), or both species (**C**). Bladders were fixed, sectioned and immunostained using antibodies to detect fibrinogen (magenta), *E. faecalis* 3143 (green) and *P. mirabilis* HI4320 (red), and DAPI was used to detect cell nuclei (blue). (**D**) Catheter segments were removed from a subset of mice 24 hpi with *E. faecalis* 3143 (top row), *P. mirabilis* HI4320 (middle row), or both species (bottom row). (**E**) Quantification of *E. faecalis* 3143 and *P. mirabilis* HI4320 co-localization with fibrinogen during single-species infections. (**F**) Quantification of co-localization of *E. faecalis* 3143 and *P. mirabilis* HI4320 during co-infections. Catheter segments were stained for fibrinogen (blue) and/or the respective pathogen (*E. faecalis* in green and *P. mirabilis* in red). Values represent the means ± SEM derived from co-localization of the catheter segments. The white broken line separates the bladder lumen (L) from the urothelium (U).

We previously determined that *E. faecalis* and *P. mirabilis* strains isolated from catheterized human subjects can establish polymicrobial colonization in a murine model of CAUTI [34]. However, co-localization during infection was not directly assessed. We therefore sought to determine whether *E. faecalis* and *P. mirabilis* co-colonize catheter segments in a murine model of CAUTI, and whether they co-localize within the catheterized bladder. Female C57BL/6J mice were inoculated by transurethral instillation of either *E. faecalis* strain 3143 or OG1RF, *P. mirabilis* strain HI4320, or a 50:50 mixture of both species, and a segment of silicone catheter tubing was inserted into the bladder at the time of inoculation. Twenty-four hours post-inoculation (hpi), mice were euthanized and the catheter, bladder, kidneys, spleen, and heart were removed for quantification of bacterial burden (Figure 2). Additionally, catheterized bladders were removed from a subset of mice and sectioned for immunohistochemistry (Figure 3), and a subset of catheters retrieved from infected mice were used for direct immunofluorescence staining to assess co-localization of both pathogens (Figure 3).

Single-species infections of *P. mirabilis* HI4320 alone and *E. faecalis* 3143 alone exhibited comparable catheter, bladder, and kidney colonization, although only *P. mirabilis* was capable of disseminating to the bloodstream to colonize the spleen and heart (Figure 2A). Both species were also capable of co-colonizing the catheter segment and bladder during co-infection (Figure 2B–D), and co-infection also appeared to facilitate dissemination of *E. faecalis* to the spleen by 24 hpi (Figure 2C), which is consistent with our prior observations at 96 hpi in CBA/J mice [34]. Notably, *E. faecalis* strain 3143 exhibited similar colonization patterns as the well-characterized strain OG1RF (Figure 2A,D). 

To determine whether *E. faecalis* and *P. mirabilis* co-localize during infection, catheterized bladders were removed from a subset of mice and sectioned for immunohistochemistry (Figure 3). In sections taken from single-species infection, *E. faecalis* colonization was exquisitely localized to areas within the bladder that contained fibrinogen (Figure 3A). In contrast, *P. mirabilis* was able to colonize areas with and without fibrinogen (Figure 3B). However, during co-infection, *P. mirabilis* largely co-localized to areas with both *E. faecalis* and fibrinogen (Figure 3C). Taken together, these data are consistent with the body of literature describing the importance of fibrinogen for facilitating *E. faecalis* colonization during CAUTI [36,37], and suggest that *P. mirabilis* may preferentially localize to fibrinogen-bound *E. faecalis* in the bladder. 

*E. faecalis* and *P. mirabilis* are both known to form persistent biofilm communities on urinary catheters. To explore biofilm formation during infection, *E. faecalis* and *P. mirabilis* co-localization and their interaction with fibrinogen were further evaluated on catheter segments retrieved from mice 24 hpi (Figure 3D and E). During single-species infection, both *E. faecalis* and *P. mirabilis* appeared to predominantly adhere to sections of the catheter where fibrinogen was present. Indeed, 100% of the adherent *E. faecalis* population exhibited co-localization with fibrinogen and ~60% of the adherent *P. mirabilis* population (Figure 3E), indicating that this host protein likely facilitates adherence and catheter biofilm formation for both species. Importantly, *E. faecalis* and *P. mirabilis* readily co-localized on catheter segments removed from coinfected mice; 100% of the *E. faecalis* population exhibited co-localization with *P. mirabilis,* and ~75% of the *P. mirabilis* population exhibited co-localization with *E. faecalis* (Figure 3D,F). Thus, these two bacterial species appear to be capable of forming polymicrobial catheter biofilms, which may influence both persistence and disease severity during CAUTI. 

### 2.3. Co-culture of Enterococcus faecalis with Proteus mirabilis Enhances Biofilm Biomass in a Contact-Dependent Manner

**Figure 4:**
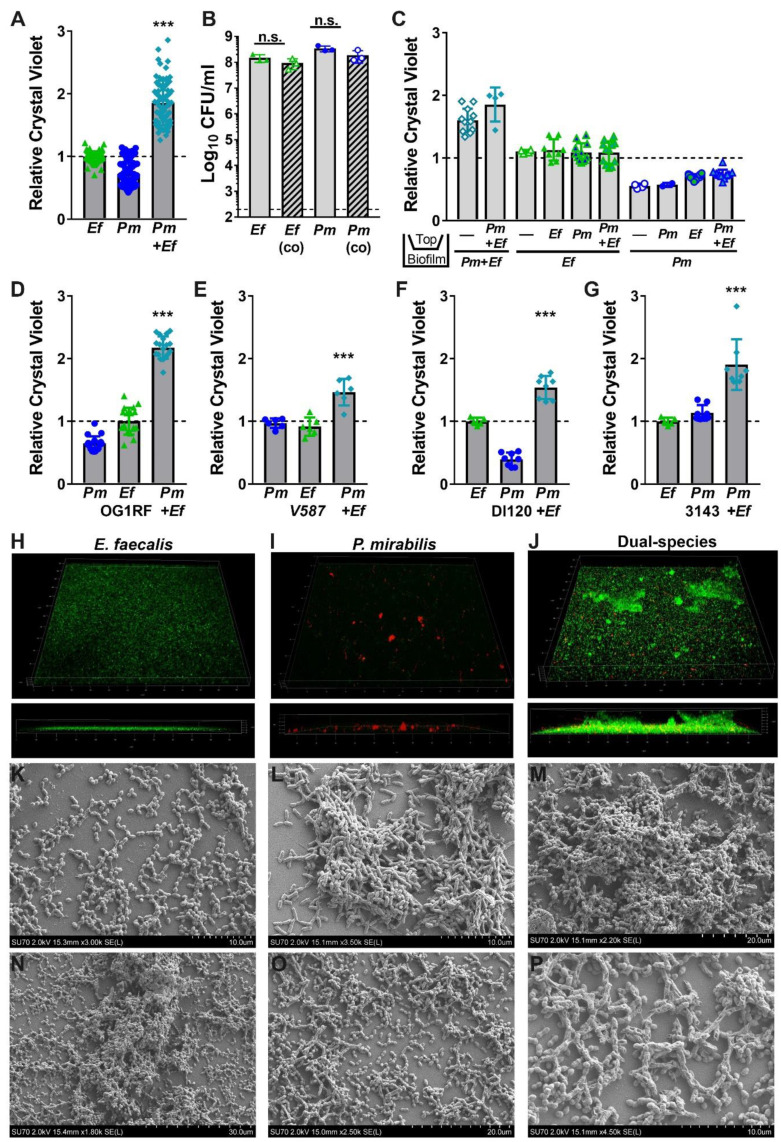
Co-culture of *E. faecalis* with *P. mirabilis* enhances biofilm biomass in a contact-dependent manner. Static biofilms were established in TSB with 10 mM glucose in 24 well tissue-culture plates (**A–G**), optically clear 24 glass-bottom plates (**H–J**), or on glass coverslips (**K–P**). All biofilms were established for 20 h unless otherwise indicated. (**A,C–G**) Biofilm biomass was quantified using crystal violet staining, and normalized to the absorbance of crystal violet at OD_570_ for biofilms formed by *E. faecalis.* (**B**) Viable biofilm-associated bacteria were quantified by plating to determine log_10_ CFU/mL of each species. (**C**) *E. faecalis* and *P. mirabilis* were physically separated by a transwell filter, and biofilms formed in the lower compartment were stained with crystal violet as above. Data represent the mean ± standard deviation for at least three independent experiments with at least two replicates each. ^ns^ = non-significant, ***P < 0.001 by Student’s *t* test. (**H–J**) Confocal microscopy of biofilms established with *E. faecalis* expressing GFP (H), *P. mirabilis* expressing DsRedDsRed (I), or both species (J). Top panels display a 3D rendering from representative z-stacks of each culture, and bottom panels display a z-slice view through the biofilm. (K–P) Representative scanning electron micrographs of biofilms formed by *E. faecalis* (**K**), *P. mirabilis* (**L**), or both species (**M–P**).

Based on the observations that *E. faecalis* and *P. mirabilis* co-colonize catheterized nursing home residents and co-localize during experimental CAUTI, including on catheter segments, we hypothesized that polymicrobial interactions between these organisms may promote the development of persistent biofilm communities. To begin testing this hypothesis, single-species and polymicrobial biofilms were established during stationary incubation in laboratory medium (TSB with 10 mM glucose), and biomass was assessed by crystal violet staining (Figure 4). Under these experimental conditions, *E. faecalis* 3143 biofilms exhibited slightly greater biomass than those formed by *P. mirabilis* HI4320, while co-culture of *E. faecalis* with *P. mirabilis* resulted in a dramatic increase in total biofilm biomass (Figure 4A). 

To explore the significance of enhanced biofilm formation during co-culture, we first sought to determine the source of the increased biomass. We previously demonstrated that broth co-culture of *E. faecalis* and *P. mirabilis* does not impact the viability or growth of either species in vitro [34]. However, enhanced biomass during stationary incubation could be a result of increased bacterial viability within the biofilm. We therefore quantified the number of colony forming units (CFUs) of each species during biofilm formation (Figure 4B). Notably, the increase in biofilm biomass was not due to differences in bacterial viability, as ~10^8^ CFUs of each organism were recovered from both single and co-culture biofilms. This indicates that increases in biofilm biomass are not due to differences in cell number, but could result from an increase in the secretion of extracellular polymeric substances (EPS) associated with the biofilm. 

We previously determined that *P. mirabilis* isolates respond to *E. faecalis* secreted factors, resulting in increased urease activity and cytotoxicity to host cells [34]. To determine whether the increase in biofilm biomass is similarly mediated by secreted factors, crystal violet biofilm quantification was conducted using a transwell system in which *P. mirabilis* and *E. faecalis* were physically separated by a permeable polycarbonate membrane (Figure 4C). When physically separated by the permeable transwell membrane, neither *E. faecalis* nor *P. mirabilis* exhibited enhanced biofilm formation, indicating that the polymicrobial interactions that increase biofilm formation are contact-dependent and not mediated by secreted factors. 

We next sought to determine whether biofilm enhancement was unique to strains HI4320 and 3143, or a generalizable phenotype for co-culture of *E. faecalis* with *P. mirabilis.* Biofilms were therefore established during co-culture of *P. mirabilis* HI4320 with *E. faecalis* OG1RF (Figure 4D) or *E. faecalis* V587 (Figure 4E), and during co-culture of *E. faecalis* 3143 with *P. mirabilis* DI120 (Figure 4F) or *P. mirabilis* 3143 (Figure 4G). Minor differences in total biomass were noted between strains, but co-culture resulted in a statistically significant increase in biofilm biomass for all combinations tested. Enhancement of biofilm formation is therefore likely a general feature that results from co-culture of numerous *P. mirabilis* strains with *E. faecalis* strains. 

### 2.4. Enterococcus faecalis and Proteus mirabilis Co-culture Alters Biofilm Architecture

Considering that co-culturing *E. faecalis* and *P. mirabilis* enhances biofilm biomass in a contact-dependent manner without an impact on bacterial CFUs, the impact of co-culture EPS was explored using confocal microscopy and scanning electron microscopy (SEM). In TSB-G, *E. faecalis* formed a monolayer with limited visible 3D architecture during single-species culture (Figure 4H), while *P. mirabilis* formed distinct cell aggregates (Figure 4I). Remarkably, co-culture of *E. faecalis* with *P. mirabilis* resulted in a dramatic increase in the 3D structure of the biofilm (Figure 4J). The co-culture biofilm was characterized by small aggregates of *P. mirabilis* and large clusters of *E. faecalis,* indicating that *P. mirabilis* may promote EPS production by *E. faecalis* (Figure 4J). This hypothesis was further supported by SEM; *E. faecalis* mostly formed a monolayer with little to no EPS (Figure 4K) and *P. mirabilis* formed distinct cell aggregates coated in EPS (Figure 4L), but dual-species biofilms exhibited larger clusters containing both bacterial species fully encased in EPS (Figure 4M). Similar to the co-localization observed on catheter biofilms from infected mice, dual-species biofilms formed in vitro indicate that *E. faecalis* likely forms the initial attachment layer while *P. mirabilis* preferentially localizes to *E. faecalis*. At increasing magnification, the exposed surface of the cover slips from dual-species biofilms predominantly show adherent *E. faecalis*, while *P. mirabilis* is largely contained within larger cell aggregates that also contained *E. faecalis* (Figure 4N–P). This observation may indicate that co-culture alters the attachment and localization of each species within the biofilm.

**Figure 5:**
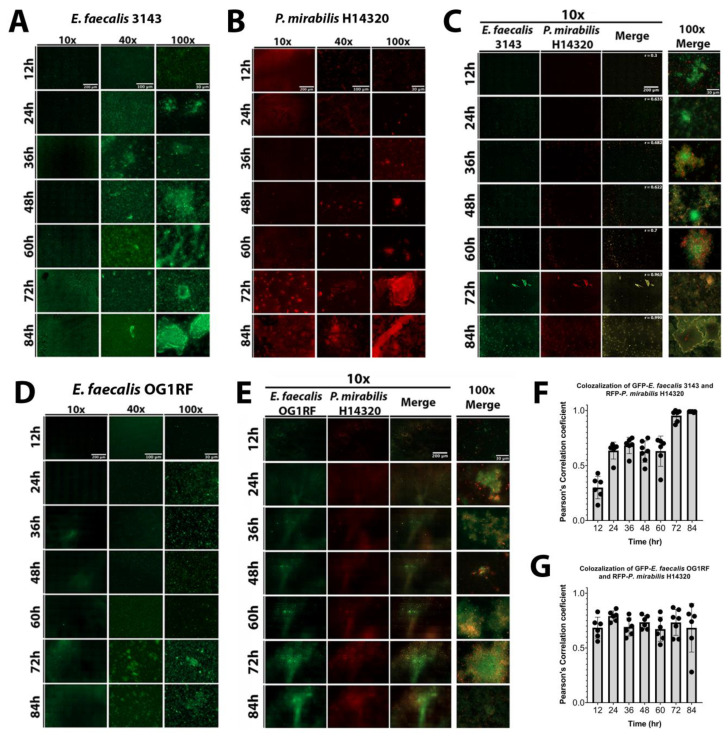
Co-culture of *E. faecalis* with *P. mirabilis* enhances biofilm 3D architecture and reduces *P. mirabilis* dispersal. Static biofilms were established on fibrinogen-coated glass-bottom petri dishes in human urine supplemented with 20 mg/mL BSA and imaged every 12 h for a total of 84 h. Images are representative of biofilms from each of the following inocula: *E. faecalis* 3143 expressing GFP (**A**), *P. mirabilis* HI4320 expressing DsRed (**B**), co-culture of *E. faecalis* 3143 GFP and *P. mirabilis* HI4320 DsRed (**C**), *E. faecalis* OG1RF GFP (**D**), and co-culture of *E. faecalis* OG1RF GFP and *P. mirabilis* HI4320 DsRed (**E**). Single-species biofilms are displayed at 10x, 40x, and 100x magnification, and co-culture biofilms are displayed at 10x magnification for each individual channel, and both 10x and 100x magnification for merged images. Concurrence and co-localization of *E. faecalis* 3143 with *P. mirabilis* HI4320 (**F**) and *E. faecalis* OG1RF with *P. mirabilis* HI4320 (**G**) were performed using Pearson’s correlation coefficient (r). The average of *r* is on the top merge panel. Values represent the means ± SD derived from co-localization at each time point.

The dynamics of biofilm formation and maturation were further examined by fluorescent microscopy. Importantly, this investigation was conducted in human urine and included the use of a fibrinogen-coated surface to facilitate *E. faecalis* attachment and growth. *E. faecalis* formed a monolayer with limited 3D architecture during the first 24 h of biofilm development, but cell aggregates were apparent by 36 hpi and biomass steadily increased out to 84 hpi (Figure 5A). *P. mirabilis* biofilms had visible aggregates during the entire time course, which were most prominent after 48 hpi, as well as crystalline structures that could be readily observed by 72 and 84 hpi (Figure 5B). *P. mirabilis* single-species biofilms also appeared to experience some degree of dispersion from 24 to 36 hpi, which was followed by robust biofilm formation at later time points (Figure 5B). In dual-species biofilms, both bacterial species were present in small aggregates from 12 to 60 hpi, and they co-localized with large crystalline structures at 72 and 84 hpi (Figure 5D). Interestingly, co-culturing *E. faecalis* and *P. mirabilis* impacted the biofilm formation behaviors of both species; *P. mirabilis* was not observed to disperse from the polymicrobial biofilm formation and instead remained co-localized with *E. faecalis* and/or bound to the fibrinogen-coated surface, and *E. faecalis* formed robust 3D architecture in the polymicrobial biofilm that was not seen during single-species biofilm development. The development of polymicrobial biofilm formation between *E. faecalis* and *P. mirabilis* increased over time (Figure 5C). The concurrence of both species was analyzed by measuring the co-localization of pixel intensity of GFP (*E. faecalis*) and DsRedDsRed (*P. mirabilis*)*,* revealing that the Pearson’s correlation coefficient trended to 1 over time (Figure 5F), which is indicative of near-perfect co-localization [38].

The well-characterized *E. faecalis* strain OG1RF was again utilized to determine whether the effects of polymicrobial biofilm formation are specific to urinary tract isolates (Figure 5D). Biofilm formation by *E. faecalis* OG1RF was not as robust as *E. faecalis* 3143, although 3D architecture was evident at 72 and 84 hpi (Figure 5D). Importantly, co-culture of *E. faecalis* OG1RF with *P. mirabilis* similarly promoted *E. faecalis* 3D architecture and prevented dispersal of *P. mirabilis* from the polymicrobial biofilm (Figure 5E). Furthermore, the concurrence analysis demonstrated a high correlation of co-localization between both species at all time points (Figure 5G). Taken together, these data indicate that interactions between *P. mirabilis, E. faecalis,* and fibrinogen significantly impact biofilm development in a way that would facilitate persistent colonization, and these interactions appear to be species specific rather than strain specific.

### 2.5. Enterococcus faecalis and Proteus mirabilis Dual-species Biofilms Exhibit Enhanced Antimicrobial Resistance

The data thus far have shown that *P. mirabilis* and *E. faecalis* persistently co-colonize catheterized nursing home residents, that they co-localize within the bladder and on catheter segments, and that the interactions between these two bacterial species increases biofilm biomass. The formation of persistent bacterial biofilms on urinary catheters is a notorious problem in health care settings, particularly those involving *P. mirabilis.* Due to its potent urease enzyme, *P. mirabilis* typically forms crystalline biofilms on catheters, which can obstruct urine flow [23,25,39]. Biofilms are also known to provide protection from host defenses and antimicrobial therapy, due to a combination of factors including the physical protection provided by the EPS, potential accumulation of antibiotic-modifying enzymes in the biofilm matrix, decreased metabolic rate, and adaptive stress responses [40]. We therefore sought to determine whether dual-species biofilms confer additional antimicrobial protection to either *P. mirabilis* or *E. faecalis*. 

**Figure 6:**
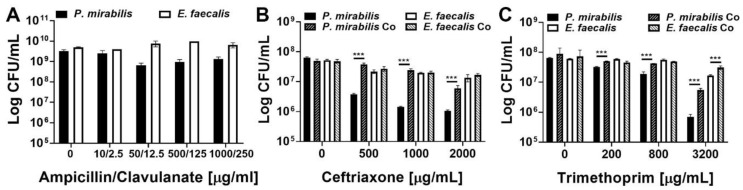
*E. faecalis* and *P. mirabilis* polymicrobial biofilms provide additional antimicrobial resistance to both species. Static biofilms were established on 24 well plates in TSB-G for 20 h, then treated with the following antimicrobials for 24 h: ampicillin-clavulanate (**A**), ceftriaxone (**B**), or trimethoprim (**C**). Data represent the mean log_10_ CFU/mL ± SD of *P. mirabilis* HI4320 (black) or *E. faecalis* 3143 (white) for single-species biofilms (solid bars) or co-culture biofilms (diagonal lines) from three independent experiments. ***P < 0.001 by Student’s *t* test.

The minimal inhibitory concentration of several antimicrobial agents utilized in the empiric treatment of UTI was first determined for each bacterial species during 18 h of planktonic growth in TSB-G (Table 1). *E. faecalis* 3143 exhibited a high level of resistance to the majority of antimicrobial agents, while *P. mirabilis* HI4320 was more sensitive to ampicillin, ceftriaxone, and trimethoprim. Ampicillin, ceftriaxone, and trimethoprim were therefore chosen to explore the level of resistance provided by the biofilm mode of growth, and clavulanate was included with ampicillin treatment to ensure that any observed resistance is strictly due to the biofilm mode of growth and not to production of a beta-lactamase (Figure 6). 

*E. faecalis* and *P. mirabilis* single-species biofilms were both highly resistant to all three antimicrobial agents, even at concentrations 50–1000 times greater than the planktonic inhibitory concentrations and well above what could be clinically achieved. Treatment with up to 1000 µg/mL of ampicillin and 250 µg/mL of clavulanate had minimal impact on the viability of single-species biofilms (Figure 6A). The impact of co-culture and polymicrobial biofilm formation was therefore not assessed for this antimicrobial combination. *E. faecalis* single-species biofilms were also highly resistant to ceftriaxone and exhibited less than a 1-log decrease in viability even after treatment with 2000 µg/mL, while *P. mirabilis* exhibited a concentration-dependent decrease in viability that was readily apparent at 500 µg/mL and greater (Figure 6B). Importantly, formation of a polymicrobial biofilm with *E. faecalis* significantly protected *P. mirabilis* from ceftriaxone at all tested concentrations, and allowed for maintenance of ~10^7^ CFU/mL of *P. mirabilis* even when treated with up to 1000 µg/mL of ceftriaxone (Figure 6B). A similar trend was observed for trimethoprim, with *E. faecalis* single-species biofilms exhibiting greater resistance than *P. mirabilis* biofilms, but formation of a polymicrobial biofilm provided protection to both species at 3200 µg/mL (Figure 6C). Thus, while *E. faecalis* and *P. mirabilis* both become highly resistant to antimicrobial agents during the biofilm mode of growth, these bacterial species readily form polymicrobial biofilms that provide additional protection and contribute to persistent colonization of catheterized hosts.

### 2.6. Co-culture of Enterococcus faecalis with Morganella morganii also Enhances Biofilm Biomass

**Figure 7:**
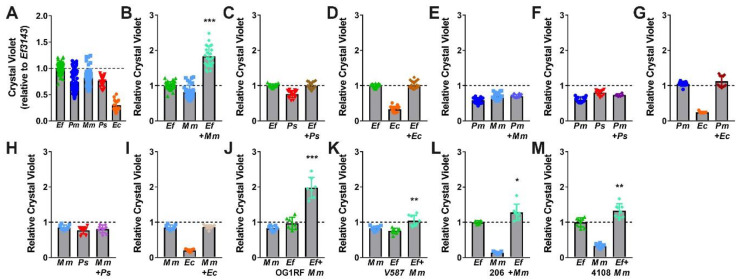
*E. faecalis* and *M. morganii* polymicrobial biofilms exhibit enhanced biomass. Static biofilms were established in TSB with 10 mM glucose in 24 well tissue-culture plates for *E. faecalis* 3143 (*Ef*), *P. mirabilis* HI4320 (*Pm*), *M. morganii* TA43 (*Mm*), *P. stuartii* BE2467 (*Ps*), and *E. coli* CFT073 (*Ec*) and all co-culture combinations with *E. faecalis, P. mirabilis,* or *M. morganii. M. morganii* isolates 206 and 4108 and *E. faecalis* isolates OG1RF and V587 were also utilized where indicated. All biofilms were established for 20 h unless otherwise indicated. Biofilm biomass was quantified using crystal violet staining and normalized to the absorbance of crystal violet at OD_570_ for single-species biofilms formed by *E. faecalis.* Data represent the mean ± standard deviation for at least three independent experiments with at least two replicates each. *P < 0.05, **P < 0.01, and ***P < 0.001 by Student’s *t* test.

While *E. faecalis* and *P. mirabilis* were the most common and persistent co-colonization pair in our recent study [35], other combinations are also frequently reported. In addition to *E. faecalis* and *P. mirabilis,* the most common organisms typically identified during polymicrobial catheter-associated bacteriuria and CAUTI include *Escherichia coli, Providencia stuartii,* and *Morganella morganii.* We therefore explored biofilm formation with these three species, as well as the impact of co-culture with *E. faecalis* and *P. mirabilis* (Figure 7). All species were capable of forming stationary biofilms in TSB-G, although biofilm formation by *E. coli* strain CFT073 was minimal (Figure 7A). Interestingly, co-culture of *E. faecalis* with *M. morganii* resulted in a similar degree of biofilm enhancement as observed for co-culture of *E. faecalis* with *P. mirabilis,* (Figure 7B). This is particularly notable as none of the other co-culture combinations resulted in biofilm enhancement (Figure 7C–I).

We again sought to determine whether biofilm enhancement was unique to co-culture of *E. faecalis* strain 3143 and *M. morganii* strain TA43, or a generalizable phenotype for co-culture of *E. faecalis* with *M. morganii.* Biofilms were therefore established during co-culture of *M. morganii* TA43 with *E. faecalis* OG1RF (Figure 7J) or *E. faecalis* V587 (Figure 7K), and during co-culture of *E. faecalis* 3143 with *M. morganii* 206 (Figure 7L) or *M. morganii* 4108 (Figure 7M). Similar to co-culture of *P. mirabilis* with *E. faecalis,* significant biofilm enhancement was observed for all strain combinations, indicating that enhancement is likely a general feature that results from co-culture of numerous *M. morganii* strains with *E. faecalis* strains. 

## 3. Discussion

Biofilm formation has long been recognized to contribute to bacterial pathogenesis and antimicrobial resistance, including a role for catheter biofilms in CAUTI [16,41,42,43]. The high incidence of polymicrobial bacteriuria during long-term catheterization has also been widely reported for decades [1,2,17]. However, few studies have examined the composition of polymicrobial bacteriuria or the impact of polymicrobial interactions on catheter biofilm formation, bacterial persistence, or likelihood of developing symptomatic infection and severe disease. In this study, we provide direct evidence from human subjects that *E. faecalis* and *P. mirabilis* frequently and persistently co-colonize catheterized nursing home residents. Furthermore, we demonstrate for the first time that these bacterial species co-localize within biofilm communities in vitro and during infection in a murine model of CAUTI, resulting in enhanced persistence and protection from antimicrobial agents in a contact-dependent manner. The interaction between these species appears to be largely unique, as the only other combination that resulted in biofilm enhancement was *E. faecalis* with a close relative of *P. mirabilis, M. morganii.*

It is notable that the time-course biofilm experiments indicate that *E. faecalis* may prevent *P. mirabilis* dispersal from developing biofilms, resulting in formation of a more robust biofilm matrix and overall biomass and facilitating the development of 3D biofilm architecture by *E. faecalis*. The polymicrobial biofilm provides additional antimicrobial protection to both bacterial species, but the effect was greatest for *P. mirabilis.* Considering that *E. faecalis* is often dismissed as a urine culture contaminant [44,45], the role of this bacterium in promoting the persistence and pathogenic potential of other bacterial species is highly significant and warrants further investigation. 

The impact of *E. faecalis* and *P. mirabilis* co-culture on antimicrobial susceptibility also has important clinical implications. *Enterococcus* species exhibit intrinsic resistance to β-lactam antibiotics and can acquire resistance to aminoglycosides [43], and *Proteus* species exhibit intrinsic resistance to tetracyclines and polymyxins, with other resistances on the rise, including aminoglycosides, fluoroquinolones, extended-spectrum β-lactam antibiotics, and carbapenems [46,47,48,49,50]. Polymicrobial biofilm formation by *E. faecalis* and *P. mirabilis* therefore severely limits the antimicrobial agents that can be used to effectively treat any resulting infections, especially considering that both bacterial species have been detected on catheters even after antimicrobial treatment eradicates viable bacteria from the urine [16]. As *E. faecalis* colonization typically precedes *P. mirabilis,* it may be advantageous to identify high-risk individuals and aggressively target *E. faecalis* to reduce the risk of developing *P. mirabilis* colonization and CAUTI sequelae. However, additional prospective investigation of bacteriuria and catheter colonization dynamics would be necessary to identify high-risk individuals and to incorporate assessment of the role of other co-colonizing organisms in developing of persistent colonization, antimicrobial resistance, and CAUTI sequelae.

## 4. Materials and Methods 

### 4.1. Urine Colonization Study Design and Participants

All study participants (or approved decision makers) provided written informed consent prior to initiation of investigation, and all participants also assented to being in the study. This study was conducted in accordance with the Declaration of Helsinki, and the protocol was approved by the University at Buffalo Institutional Review Board (STUDY00002526). The data presented in this manuscript were collected through a study conducted at two nursing homes located in Buffalo, New York between July 2019 and March 2020. Full experimental details of the study and patient characteristics are provided in a separate preprint [35]. Briefly, inclusion criteria were a) residence at a participating nursing facility; b) presence of an indwelling urinary catheter (Foley or suprapubic) for at least 12 months; c) ≥21 years of age; and d) informed consent from the resident or approved decision maker. Residents receiving end-of-life care were excluded. Participants were visited by a study team member and licensed practicing nurse weekly for up to 30 weeks, and a urine sample was collected from the catheter port at each visit using sterile technique. Urine cultures were stored at 4 °C for no more than 4 h prior to culturing for bacterial isolation. *Enterococcus* isolates were identified by colony morphology and hemolysis characteristics on Columbia CNA agar, PYR activity, and lack of catalase activity, and were confirmed to the species level using previously-described primer sets [51]. *Proteus* isolates were identified by colony morphology on MacConkey plates and swarming on blood agar, and were identified to the species level using API-20E test strips (BioMérieux, Marcy-l’Étoile, France).

### 4.2. Urine Collection for Use in Biofilm Time-Course Microscopy Experiments

All participants signed an informed consent form, urine collection was conducted in accordance with the Declaration of Helsinki, and collection protocols were approved by the local Internal Review Board at the University of Notre Dame under study #19-04-5273. Human urine was collected and pooled from at least two healthy female donors between 20 and 35 years of age. Donors had no history of kidney disease, diabetes or recent antibiotic treatment. Urine was sterilized using a 0.22 μm filter (Sigma Aldrich) and pH normalized to 6.0–6.5 prior to use. When supplemented with Bovine Serum Albumin (BSA), sterile urine was filter sterilized again following BSA addition. For fibrinogen supplementation, fibrinogen was added directly to the sterile urine and not filtered again. 

### 4.3. Bacterial Strains

*Proteus mirabilis* strains HI4320 and DI120, *Providencia stuartii* BE2467, and *Morganella morganii* TA43 were isolated from the urine of catheterized patients from a chronic care facility in Maryland [25]. *Enterococcus faecalis* 3143, *P. mirabilis* 3143, and *M. morganii* 4108 were isolated from the urine of catheterized nursing home residents in Michigan [11], and *M. morganii* 206 was isolated from the urine of a catheterized nursing home resident at a facility in New York [35]. *Escherichia coli* CFT073 was isolated from a patient hospitalized for acute pyelonephritis [52]. *E. faecalis* V587 was isolated from the urine and blood of a hospitalized patient in Missouri [53], and *E. faecalis* OG1RF was isolated from the mouth of a human subject [54,55,56]. *P. mirabilis* DsRed refers to *P. mirabilis* HI4320 with pGEN-MCS encoding a red fluorescent protein [57]. 

A new cloning vector was designed to introduce green fluorescent protein (GFP) into *E. faecalis* strains 3143 and OG1RF. This plasmid, named pAOJ20, contains the following components: 1) pAMβ1 origin of replication and the P_ermB_-GFP cassette from plasmid pTRKH3-ermGFP (Addgene plasmid 27169, deposited by Michela Lizier) [58]; 2) the chloramphenicol resistance cassette from pIMAY (Addgene plasmid 68939, deposited by Tim Foster) [59]; and 3) the *rrnB* T1-T2 terminator region and p15a origin of replication from pGEN-MCS. Using Q5 High Fidelity Polymerase (NEB), PCR fragments containing the pAMβ1 origin of replication and the P_ermB_-GFP cassette were amplified from pTRKH3-ermGFP using primer sets AOJ_424/AOJ_425, and AOJ_426/AOJ_427, the *rrnB* T1-T2 terminator region and p15a origin of replication were amplified from pGEN-MCS with primer sets AOJ_428/AOJ_429 and AOJ_432/AOJ_433, and the chloramphenicol resistance cassette from pIMAY was amplified with primer set AOJ_430/AOJ_431. The PCR fragments were assembled into a circular plasmid using NEBuilder HiFi DNA Assembly Master Mix (NEB) and transformed into Top 10 *E. coli*. The resulting vector, pAOJ20, can be used transferred between *E. coli* and Gram-positive bacteria, constitutively expresses GFP, can be selected for in *E. coli* and *E. faecalis* using chloramphenicol at a concentration of 20 µg/mL, and contains unique BsaI cut sites surrounding most plasmid features allowing for simple swapping of plasmid elements. The sequences of the primers used in generation of pAOJ20 are provided in Table 2. Plasmid pAOJ20 was isolated from Top 10 *E. coli* using a ZymoPURE II Plasmid Maxiprep Kit (Zymo Research) according to the manufacturer’s protocol, and transformed into *E. faecalis* strains. Briefly, competent *E. faecalis* cells were electroporated to introduce pAOJ20, and transformants selected on chloramphenicol and verified for expression of GFP by microscopy. 

### 4.4. Bacterial Culture Conditions

*Proteus mirabilis* HI4320 was cultured at 37 °C with aeration in 5 mL of LB broth (10g/L tryptone, 5g/L yeast extract, 0.5g/L NaCl) or on LB broth solidified with 1.5% agar. *Enterococcus faecalis* 3143 and OG1RF were cultured in BHI broth (Difco) at 37 °C without aeration or on BHI agar (Difco). Biofilm assays were performed using Tryptic Soy Broth (Research Products International) supplemented with 10 mM glucose (TSB-G) or filter-sterilized human urine. 

### 4.5. In Vivo Mouse Model

The University of Notre Dame Institutional Animal Care and Use Committee approved all mouse infections and procedures as part of protocol number 18-08-4792MD. All animal care was consistent with the Guide for the Care and Use of Laboratory Animals from the National Research Council [4]. Mice used in this study were ~6-week-old female wild-type C57BL/6J mice purchased from Jackson Laboratory. Mice were subjected to transurethral implantation and inoculated as previously described [3]. Briefly, mice were anesthetized by inhalation of isoflurane and implanted with a 6-mm-long silicone catheter. Mice were infected immediately following catheter implantation with 50 μl of ∼2 × 10^7^ CFU/mL in PBS, of one of the bacteria strains or a co-culture, made by mixing equal amounts of the single cultures of each strain, introduced into the bladder lumen by transurethral inoculation. To harvest the catheters and organs, mice were sacrificed at 24 h post-infection by cervical dislocation after anesthesia inhalation; the silicone catheter, bladder, kidneys, heart and spleen were aseptically harvested for CFU enumeration. Catheters were either subjected to sonication for CFU enumeration or fixed for imaging as described below. Bladders for immunofluorescence and histology analysis were fixed and processed as described below. Co-infection homogenates were plated on both LB and BHI plates with the corresponding antibiotics.

### 4.6. Antibodies

Primary antibodies: goat anti-fibrinogen (Sigma-Aldrich), mouse anti-EbpABC (1:1000) for *E. faecalis* [43] or rabbit anti-*E.coli* serotype O/K (Invitrogen) (1:100) for *P. mirabilis* [60]. Secondary antibodies: Alexa Fluor 488-labeled donkey anti-mouse, Alexa Fluor 594-labeled donkey anti-rabbit, Alexa Fluor 647-labeled donkey anti-goat, donkey anti-goat IRDye 800CW, donkey anti-goat IRDye 680LT, donkey anti-rabbit IRDye 680LT, or donkey anti-mouse IRDye 800CW were used. Alexa Fluor secondary antibodies were purchased from Invitrogen Molecular Probes, and IRDye conjugate secondary antibodies were from LI-COR Biosciences.

### 4.7. Immunofluorescence

Bladders were fixed in neutral buffered formalin for 24 h at room temperature and dehydrated in 70% ethanol overnight at 4 °C. Fixed bladders were embedded in paraffin, sectioned, and mounted on slides. Bladder sections were deparaffinized with xylene (two times for 10 min), rehydrated with isopropanol (three times for 5 min), and washed with water for 5 min. Bladder antigens were retrieved by boiling the section in 10 mM Na-citrate for 30 min and washed in water for 5 min, followed by PBS washes (three times for 5 min). The sections were then blocked with 1% BSA and 0.3% Triton X-100 in PBS for 1 h and incubated with primary antibodies (1:100) overnight at 4 °C, followed by three washes with PBS. Next, sections were incubated with the Alexa Fluor secondary antibodies (1:500) for 1 h at room temperature, followed by three washes with PBS. Sections were then counterstained with Hoechst dye specific for DNA (1:20,000 in PBS). Slides were sealed with prolong gold (Invitrogen). The sections were analyzed by epifluorescence microscopy on a Zeiss Axioskop 2 MOT Plus microscope.

### 4.8. Catheter Imaging

Catheters were imaged as previously described [43]. Briefly, catheters were fixed with 10% neutralized formalin (Leica Biosystems) for 20 min immediately following harvest, washed with PBS-T (PBS containing 0.05% Tween 20 [VWR]) three times and blocked overnight at 4  °C with PBS containing 1.5% BSA containing 0.1% sodium azide (Acros Organics). Catheters were then washed with PBS-T three times and incubated with two of the indicated primary antibodies at room temperature for 2 h. Catheters were washed with PBS-T (three times) and incubated with corresponding IRDye secondary antibodies (1:50,000) (LI-COR Biosciences) for 2 h at room temperature. Lastly, implants were washed with PBS-T three times, PBS three times and then allowed to dry. The Odyssey Imaging System (LI-COR Biosciences) was used to examine the infrared signal. Controls for autofluorescence included nonimplanted catheters subjected to staining. Co-localization was determined for catheters using ImageJ plugin pixel color counter. The number of red, green, and yellow pixels on each catheter were calculated. Yellow pixels are the result of the overlap of green and red. The percent of overlap was calculated for red and green relative to the total number of pixels in that color.

### 4.9. Biofilm Formation for Determination of Viability

Biofilm formation was performed in plastic 24-well microtiter dishes (Falcon 353047) as previously described [61] with minor modifications. Inocula were normalized to ~2 × 10^7^ CFU/mL using a UV/Vis Spectrophotometer (Amersham Biosciences Ultrospec 2100 pro) as follows: OD_600_ 0.02 for *P. mirabilis,* or 0.04 for *E. coli* and *E. faecalis*. Blanks for normalization were assay medium or PBS. Static biofilms were grown in 24-well plates by introducing 10^7^ CFU into TSB-G (10 mM glucose) and incubated at 37 °C for 20 h. Assays were housed in unsealed plastic bags containing a moistened Kimwipe for the duration of biofilm formation to prevent desiccation.

Additionally, biofilms were generated in 24 well microtiter plates separating *P. mirabilis* and *E. faecalis* into apical and basolateral compartments using 6.5 mm transwell filters with a pore size of 0.33 µm (Falcon 353095). A volume of 750 uL TSB-G was added to a 24 well plate and inoculated with equal quantities of *P. mirabilis* and *E. faecalis* as described above. Where indicated, a transwell filter was then added to each well and inoculated with either *P. mirabilis* or *E. faecalis.* Plates were then incubated using conditions identical to those described above.

Following incubation, supernatants were removed and biofilms were washed twice with 1x PBS to remove any remaining planktonic bacteria. A volume of 1 mL of sterile PBS was then added to each well and biofilms were scraped with a sterile micropipette tip to resuspend viable bacteria contained within each biofilm. Samples underwent serial 10x dilutions and were spiral plated onto selective media for enumeration of CFU using a ProtoCOL 3 automated colony counter (Synbiosis). LB and BHI were supplemented with 25 µg/mL kanamycin, 20 µg/mL chloramphenicol, 25 µg/mL ampicillin, 2.5 µg/mL tetracycline, or 100 µg/mL streptomycin to distinguish between species. 

### 4.10. Crystal Violet Quantification

Following 20 h incubation and biofilm formation as above, supernatants were carefully removed from wells of a 24-well plate using a suction manifold fitted with a 300 uL tip, inserted slowly at a 45° angle while making sure to avoid touching the sides and bottom of wells. Biofilms were washed twice with PBS, fixed for 15 min with 1 mL of ice-cold 99% ethanol, and air dried for 30 min in a fume hood. Biofilms were then stained with 1 mL 0.1% crystal violet for 60 min, washed with once with distilled H_2_O to remove excess stain, and solubilized in 1 mL of 95% ethanol for 15 min on an orbital shaker at 220 rpm. Crystal violet absorbance was measured at 570 nm using a microplate reader (BioTek Synergy H1 Microplate Reader), and absorbance was blanked using two cell-free control wells for each biofilm assay.

### 4.11. Confocal Laser Scanning Microscopy

Biofilms were grown in 24-well optically clear glass-bottom plates (Mattek) using the conditions described above. Biofilms were then washed with PBS, fixed using 4% Glutaraldehyde for 1 h at ambient temperature, and supernatants were replaced with a 1:50 dilution of ProLong Gold Antifade Reagent (Thermofisher). Images were acquired on a Leica SP8 TCS confocal laser scanning microscope (Lecia Microsystems, Buffalo Grove, IL, USA).

### 4.12. Scanning Electron Microscopy

Biofilms were established on glass coverslips as described above. Biofilms were washed in PBS and fixed for 1 h with 2.5% glutaraldehyde in 0.1 M sodium cacodylate buffer containing 0.075% ruthenium red and 0.075 M lysine acetate, pH 7.2. Samples were rinsed three times with 0.2 M sodium cacodylate buffer containing 0.075% ruthenium red (pH 7.2) and then subjected to graded incubations in 30%, 50%, 75%, 95%, and 100% ethanol. Samples were submerged twice in 100% hexamethyldisilazane and air dried. Scanning electron microscopy (SEM) images were captured with a Hitachi SU-70 microscope equipped with a tilt stage for side angle views. Bacteria were visualized by scanning electron microscopy (SEM) at the UB Instrumentation center with a Hitachi SU-70 SEM. 

### 4.13. Time-Course Biofilm Imaging

Biofilm development was monitored by using a Zeiss microscope with 10x and 40x and 100x magnification. Images were acquired using Zen Pro software (Carl Zeiss, Thornwood, NY) and processed using Zen Pro and ImageJ software. Single cultures were prepared by normalizing the OD_600_ of overnight cultures to ~1.2 in PBS and diluting 1:100 into human urine supplemented with 20 mg/mL bovine serum albumin (BSA). The diluted urine culture was added to a fibrinogen-coated glass-bottom petri dish. Dishes were coated with 150 ug/mL fibrinogen. Co-cultures were made by mixing the two species at an OD_600_ ~1.2 at a 1:1 ratio then diluting 1:100 into BSA supplemented urine. Dishes were incubated for 84 h at 37 °C. Every 12 h, the urine was gently aspirated and the dish washed with PBS to remove planktonic bacteria prior to imaging. Drying was avoided during imaging at 40x and 100x magnification by rehydrating and aspirating PBS as needed. Then, following imaging, urine supplemented with 20 mg/mL BSA was added to the dish and incubated at 37 °C until the next time point. Co-localization analysis of the biofilm time series was performed by quantifying the pixels intensities of the GFP-*E. faecalis* and sdRed- *P. mirabilis* by using the ImageJ Coloc2 plugin. For each time point’s image, six quadrants (5 x5-grid region) were analyzed by using a random number generator. The averages at each time point were calculated and displayed in the bottom left-hand corner of the corresponding image. The point spread functions (PSF) was calculated using the equation PSF= d/pixel size where d = lambda/2*NA. Costes was used for threshold for Pearson’s regression analysis.

### 4.14. Minimum Inhibitory Concentration Assays

Antimicrobials were diluted as per manufacturer specifications and stored at -20 °C until needed, and included trimethoprim (Cayman Chemical 16473), ceftriaxone sodium salt (Cayman Chemical 18866), nitrofurantoin (TCI Chemicals N0883), ampicillin sodium salt (Research Products International A40040), and daptomycin (Cayman Chemical 15651). For planktonic bacteria, the minimum inhibitory concentration was determined as to be the lowest concentration that impaired visible growth during an 18 h incubation in TSB-G at 37 °C with aeration. Growth was also assessed by OD_600_ measurements taken every 15 min for 18 h as previously described using a microplate spectrophotometer (Biotek Synergy H1) [62]. Susceptibility of biofilms to antimicrobials was determined by establishing biofilms as above. After 20 h, supernatants were carefully removed and replaced with fresh TSB-G containing antimicrobials. Biofilms were then incubated for an additional 24 h, and viable CFUs/mL were determined as above. 

### 4.15. Statistical Analysis

Study participant characteristics were analyzed by chi-square tests, t test, and exact logistic regression using StataIC 15.1 (StatCorp, College Station, Texas). The significance of experimental results was assessed by two-way analysis of variance (ANOVA), unpaired Student’s *t* test, or Mann–Whitney *U* test, as indicated in the figure legends using GraphPad Prism, version 7.03 (GraphPad Software, San Diego, CA, USA).

## Figures and Tables

**Table 1:** Minimum inhibitory concentrations of antimicrobials against *E. faecalis* 3143 and *P. mirabilis* HI4320 cultured in TSB-glucose.

	*P. mirabilis* HI4320	*E. faecalis* 3143
**Ampicillin**	16	32
**Ceftriaxone**	4	>64
**Daptomycin**	>256	>256
**Trimethoprim**	32	>64
**Nitrofurantoin**	128	128

Values represent the lowest antimicrobial concentration (µg/mL) at which no visible bacterial growth was observed after an 18 h incubation in TSB-G at 37 °C with aeration across three independent experiments.

**Table 2:** Primer Sequences for generation of pAOJ20.

Name	Sequence
AOJ_424	ACACTAGGCCCGGTCTCCCAAGAATTAGAAATGAGTAGAT
AOJ_425	ATCGATACCGAGACCTTCTATTTAATCACTTTGACTAGCA
AOJ_426	CCTAGTGTTTTAGGAGACCGAGCCACTATCGACTACGC
AOJ_427	TGATTAAATAGAAGGTCTCGGTATCGATAAGCTTAGTCTA
AOJ_428	CGATAGTGGCTCGGTCTCCTAAAACACTAGGCCCAAGA
AOJ_429	CTGCGGTCTCATGGTCCATGCGAGAGTAGGGAACT
AOJ_430	CGCATGGACCATGAGACCGCAGGTTAGTGACATTAGAA
AOJ_431	CTCATTATTTGGGCAGGAGACCCTTTAGTGAGGGTTAATT
AOJ_432	CTAAAGGGTCTCCTGCCCAAATAATGAGCTAGCCCG
AOJ_433	TCTAATTCTTGGGAGACCGGGCCTAGTGTTTTAGATCC
